# Serum PAPP-A as a potential biomarker for early detection of ectopic pregnancy: an exploratory case-control study

**DOI:** 10.3389/frph.2026.1891223

**Published:** 2026-07-22

**Authors:** Xiaoyan Sun, Ning Wang, Zongtao Liu, Hang Lu, Xiaohui Yang, Mingming Ma, Haixia Liu

**Affiliations:** 1 Department of Gynecology, Affiliated Qingdao Third People’s Hospital, Qingdao University, Qingdao, China; 2 Department of Clinical Laboratory, Affiliated Qingdao Third People’s Hospital, Qingdao University, Qingdao, China

**Keywords:** biomarker, ectopic pregnancy, exploratory study, LIF, PAPP-A, VEGF

## Abstract

**Introduction:**

In this exploratory case-control study, we investigated the potential associations of pregnancy-associated plasma protein-A (PAPP-A), β-human chorionic gonadotrophin (β-HCG), vascular endothelial growth factor (VEGF), and leukemia inhibitory factor (LIF) with ectopic pregnancy (EP), using logistic regression to describe unadjusted associations and receiver operating characteristic (ROC) curve analysis to characterise exploratory biomarker performance.

**Methods:**

We conducted a case-control study including 48 patients with EP and 46 patients with normal intrauterine pregnancy as controls, recruited between October 2022 and May 2024 at a single center. Logistic regression was applied to describe associations between each biomarker and EP. ROC curves were derived from these models to explore discrimination, and an exploratory threshold was identified using the Youden index.

**Results:**

The area under the curve (AUC) for β-HCG and PAPP-A were the highest (0.85 and 0.84, respectively), compared with VEGF (0.73) and LIF (0.54). PAPP-A showed the strongest association among the candidate markers (OR = 0.774), with an exploratory sensitivity of 0.77. VEGF had the highest specificity (0.96). The combined exploratory AUC of PAPP-A and β-HCG (0.89) was similar to that of PAPP-A and VEGF (0.88).

**Conclusion:**

This exploratory study suggests that PAPP-A may be a promising candidate biomarker for early EP detection in the context of an exploratory case-control comparison with normal intrauterine pregnancy, with performance comparable to β-HCG. Further prospective studies with external validation in clinically relevant populations are needed to confirm these preliminary findings and assess clinical potential.

## Key message

In this exploratory case-control study, PAPP-A showed performance comparable to β-HCG as a candidate biomarker for early detection of ectopic pregnancy. These preliminary findings are hypothesis-generating and require assessment in broader, prospective cohorts before any clinical application can be considered.

## Introduction

Ectopic pregnancy (EP) is a frequent condition in obstetrics and gynecology that occurs when a fertilized egg implants outside the uterus. There are several types of EPs, including fallopian tube, ovarian, abdominal, broad ligament, and cervical pregnancies. Fallopian tube pregnancies are the most prevalent among EPs. EP is a leading cause of acute abdominal issues in gynecology, accounting for over 80% of such cases (1). In cases of EP, particularly in tubal pregnancies, the embryo's development may be unsustainable, potentially causing a rupture that leads to bleeding and delays treatment. Without prompt resuscitation, this can result in shock, fainting, or death. In Europe and the United States, EPs occur in about 1%–2% of pregnancies and account for nearly 75% of early-pregnancy maternal deaths (2, 3). The number of EP cases in U.S. hospitals increased from 17,800 cases in 1970 to 88,400 in 1989. The incidence rate is 11.5/1,000 in the United Kingdom and about 10/1,000 in China (4–6).

EP is typically diagnosed using ultrasound, serum β-HCG, laparoscopy, curettage, and aspiration of the posterior vaginal fornix. While cases with clear symptoms are easily diagnosed, atypical cases pose a significant clinical challenge. Improving early detection rates is a pressing issue for obstetricians and gynecologists, which highlights the need for new non-invasive candidate markers (7–9).

β-HCG is a widely utilized serum marker for the clinical detection of EP. However, fluctuations in β-HCG levels are not consistently reliable, and reliance on β-HCG may lead to misclassification in approximately 17% of EP cases (10, 11). Abdominal EPs can result in a delayed increase in serum β-HCG levels, potentially leading to missed or delayed identification (12). Several studies have suggested PAPP-A, VEGF, and LIF as potential *in vitro* candidate markers for the early detection of EP (13–15). Early studies by Bischof et al. demonstrated that PAPP-A levels are lower in ectopic and non-viable intrauterine pregnancies than in normal pregnancies, however, a single PAPP-A measurement showed limited value for distinguishing EP from intrauterine abortion and has not yet been established as a routine clinical decision-making tool. More recently, Barnhart et al. reported that, in a multiplexed biomarker panel, PAPP-A alone showed only modest discriminatory performance, and high accuracy was achieved only when combined with other biomarkers in complex models (11). Nonetheless, the independent and combined exploratory performance of PAPP-A, VEGF, and LIF in distinguishing EP from normal intrauterine pregnancy in a Chinese population has not been systematically characterized.

This exploratory case-control study was designed to investigate potential associations between serum PAPP-A, VEGF, LIF levels and EP, and to characterize the exploratory discriminatory performance of each biomarker and their combinations, establishing a foundation for future diagnostic validation studies.

## Methods

### Study design and setting

This study was designed and reported as an observational exploratory case-control study following the STROBE (Strengthening the Reporting of Observational Studies in Epidemiology) guidelines for case-control studies (16). It was conducted at a single center (Affiliated Qingdao Third People's Hospital, Qingdao University) between October 2022 and May 2024. The study was approved by the Ethics Committee of Qingdao Third People's Hospital (Approval No. 2021Y1202097, dated December 2nd, 2021), and written informed consent was obtained from all participants. All methods were performed in accordance with relevant guidelines and regulations.

### Participants

A total of 94 participants were enrolled: 48 patients with a confirmed diagnosis of EP (case group) and 46 patients with confirmed normal intrauterine pregnancy (control group). Clinical history was collected by clinicians at the patients' initial visit, and information on smoking status, parity, body weight, and body height was recorded in the medical records.

Inclusion criteria for EP cases were: (1) history of amenorrhoea, with or without abdominal pain and vaginal bleeding; (2) no intrauterine gestational sac detected on ultrasonography, or the presence of an extrauterine gestational sac with embryonic cardiac activity; and (3) serum β-HCG levels below the normal range for the corresponding gestational age. Diagnosis was confirmed by laparoscopic surgery.

Inclusion criteria for normal intrauterine pregnancy controls were: (1) history of amenorrhoea, generally without obvious abdominal pain or vaginal bleeding, or with only minimal implantation bleeding; (2) presence of an intrauterine gestational sac on ultrasonography, with a visible yolk sac and embryonic cardiac activity within the sac; and (3) serum β-HCG levels consistent with gestational age estimated from the duration of amenorrhoea. Diagnosis was confirmed by postpartum verification of pregnancy outcome.

The gestational age in both groups was 6–8 weeks, as determined by ultrasonography or the date of the last menstrual period. Blood sampling and ultrasonographic examination were completed within 24 h of the initial visit for all participants.

Exclusion criteria encompassed: (1) use of medications that may influence test outcomes; (2) severe cardiac, hepatic, or renal insufficiency, or reproductive system tumors or other malignancies; (3) severe systemic infections, uterine abnormalities, endocrine disorders, immune system diseases, or mental disorders; and (4) recent use of hormones or immunosuppressants.

**Figure 1:**
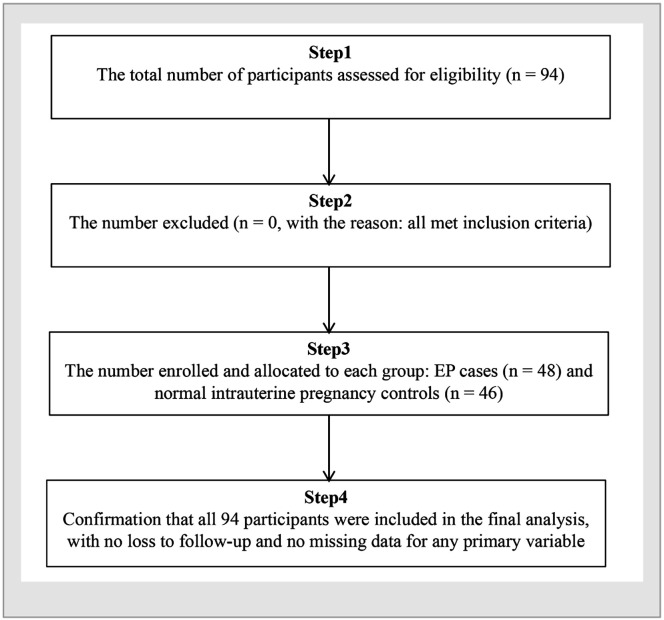
Participant flowchart. A total of 94 participants were assessed for eligibility between October 2022 and May 2024. All 94 met the inclusion criteria and none were excluded. The final analysis included 48 EP cases and 46 normal intrauterine pregnancy controls. There were no missing data for any primary analysis variable.

A total of 94 participants were assessed for eligibility. All 94 met the inclusion criteria and none were excluded, yielding 48 EP cases and 46 controls included in the final analysis. No participants were lost to follow-up or withdrew after enrolment. There were no missing data for any of the primary variables (Figure 1 for a participant flowchart).

### Variables

The primary exposure variable was the diagnosis of EP. The primary outcome of interest was the association between each serum biomarker and EP status, with the following biomarkers as the main variables of interest: serum PAPP-A (ng/mL), serum β-HCG (mIU/mL), serum VEGF (pg/mL), and serum LIF (pg/mL). Potential confounders considered were age, parity, smoking status, and body mass index (BMI, calculated as body weight in kilograms divided by height squared in meters).

### Data sources and measurement

Blood samples from all participants were obtained from the gynaecology or antenatal outpatient clinics. Venous blood collection (5 mL, fasting) was completed within 24 h of the initial consultation. Collected blood was stored at −80 °C for subsequent analyses. Detailed information on the detection reagents and methods for each biomarker is provided in Table 1. All testing was carried out by qualified personnel in certified medical laboratories.

**Table 1:** Information on potential candidate biomarker detection reagents and methods.

Biomarkers	Kit Manufacturer	Detection method	Sensitivity	Detection range	Inter-batch variation	Intra-batch variation
Human-β-HCG	Roche	Electrochemiluminescence	0.2 mIU/mL	0.2–10,000 mIU/mL	6.3%	4.2%
Human-PAPP-A	Shanghai mlbio	ELISA	0.18 ng/mL	0.36–40 ng/mL	<8%	<5%
Human-LIF	Shanghai mlbio	ELISA	15.62 pg/mL	31.20–2,000 pg/mL	<10%	<5%
Human-VEGF	Shanghai mlbio	ELISA	0.50 pg/mL	1–240 pg/mL	<10%	<5%

### Bias

Potential sources of bias in this study include selection bias and information bias. Regarding selection bias: the use of clearly defined EP cases and healthy intrauterine pregnancy controls creates an “extreme phenotype” comparison that may overestimate biomarker discriminatory performance compared with the real clinical setting. Regarding information bias: measurement errors arising from differences in assay methodology and analytical performance may affect biomarker levels. We minimized this by using standardized laboratory procedures and restricting gestational age to 6–8 weeks across both groups to reduce systematic gestational-age-related differences in biomarker levels.

### Study size

This study was conducted as an exploratory investigation; no formal *a priori* sample size calculation was performed. The achieved sample size was 48 EP cases and 46 controls (total *n* = 94). The lower limits of the AUC confidence intervals for the key biomarkers were considerably above 0.5, supporting the robustness of the findings for exploratory purposes. To contextualize the preliminary nature of these findings and inform future research, we estimated the sample size required for a robust prospective validation study targeting the PUL population [assumed EP prevalence 8% (17, 18)]: using the formula *n* = [Z^2^ × Sen ×   (1−Sen)]/L^2^, where Z = 1.96 (95% CI), Sen = 0.85 (derived from our ROC analysis), and L = 0.05 (precision), approximately 196 EP cases and 2,450 total participants would be required (19).

### Quantitative variables

All biomarker concentrations (PAPP-A, β-HCG, VEGF, and LIF) were treated as continuous variables in the logistic regression models. No arbitrary categorization was applied. Exploratory thresholds were derived *post hoc* using the Youden index (sensitivity + specificity − 1), and the value corresponding to the maximum Youden index was designated as the exploratory threshold.

### Statistical methods

Statistical analysis was conducted using R software version 4.4.2. Group differences in biomarker concentrations (β-HCG, PAPP-A, LIF, VEGF) were assessed using the Mann–Whitney *U*-test, given the non-normal distribution of these variables. Differences in categorical variables (smoking status, parity) were assessed using the chi-square (*χ*^2^) test. Age and BMI were compared using the Mann–Whitney *U*-test. A *p*-value < 0.05 was considered statistically significant.

Logistic regression models were constructed to describe univariate associations between each biomarker and EP status, and to derive predicted probabilities for ROC curve analyses. ROC curves were generated from the predicted probabilities of univariate logistic regression model, and for combined models incorporating multiple biomarkers (PAPP-A + VEGF; PAPP-A + β-HCG; PAPP-A + VEGF + LIF). All combined models were built using multivariable logistic regression with the respective biomarkers entered as continuous covariates, and the predicted probabilities from univariate model were used to generate the corresponding ROC curve. The AUC and its 95% confidence interval were calculated for each model. Differences between AUC values were assessed using the DeLong test. Exploratory thresholds were identified using the Youden index.

Given the exploratory nature of this study, all reported thresholds, performance metrics, and associations are considered hypothesis-generating and should not be interpreted as clinically validated decision thresholds or predictive tools.

## Results

### Participants

Between October 2022 and May 2024, 94 participants were assessed for eligibility and enrolled. Of these, 48 were diagnosed with EP and 46 had confirmed normal intrauterine pregnancies. All 94 participants were included in the final analysis (Figure 1).

### Descriptive data

As shown in Table 2, there were no statistically significant differences between the EP and control groups in age (median 32.0 vs. 31.5 years, *p* = 0.056), parity (*p* = 0.507), smoking status (*p* = 0.991), or BMI (median 25.6 vs. 25.4 kg/m^2^, *p* = 0.102).

**Table 2:** Baseline characteristics of the control group and patients with EP.

Characteristics	Healthy (*n* = 46) EP (*n* = 48)		*p*
Age, median (Q1, Q3)	31.5 (28.3–33.0)	32.0 (29.7–35.0)	0.056^b^
Parity, *n* (%)	1.63 (1–2)	1.55 (1–2)	0.507^a^
1	17（37）	22（46）	
2	29（63）	26（54）	
Smoking status, *n* (%)			0.99^a^
Yes (%)	2 (4)	3 (6)	
No (%)	44 (96)	45 (94)	
BMI, median (Q1, Q3)	25.4 (24.9–25.8)	25.6 (25.3–25.8)	0.102^b^
β-HCG, median (Q1, Q3) (mIU/mL)	17,885 (9,644–50,557)	3,109 (1,742–9,583)	<0.01*^,^^b^
PAPP-A, median (Q1, Q3) (ng/mL)	15.8 (11.2–18.2)	5.4 (3.8–7.6)	<0.01*^,^^b^
VEGF, median (Q1, Q3) (pg/mL)	126.5 (87.0–184.1)	187.0 (154–248.5)	<0.01*^,^^b^
LIF, median (Q1, Q3) (pg/mL)	987.1 (937.0–1,066.5)	984.0 (929.0–1,065.0)	0.565^b^

a Chi-squared test

b Mann–Whitney *U*-test.

BMI, body mass index (kg/m^2^).

* Statistically significant (*p* < 0.05).

### Outcome data

Median concentrations of β-HCG (3,109 vs. 17,885 mIU/mL), PAPP-A (5.4 vs. 15.8 ng/mL), and VEGF (187.5 vs. 126.5 pg/mL) differed significantly between the EP and control groups (*p* < 0.01). Specifically, PAPP-A and β-HCG concentrations were significantly lower in the EP group, while VEGF was significantly higher. In contrast, LIF (median 984 vs. 987.1 pg/mL, *p* = 0.565) did not differ significantly between groups (Table 2).

## Main results

### Exploratory discrimination of individual biomarkers

**Figure 2:**
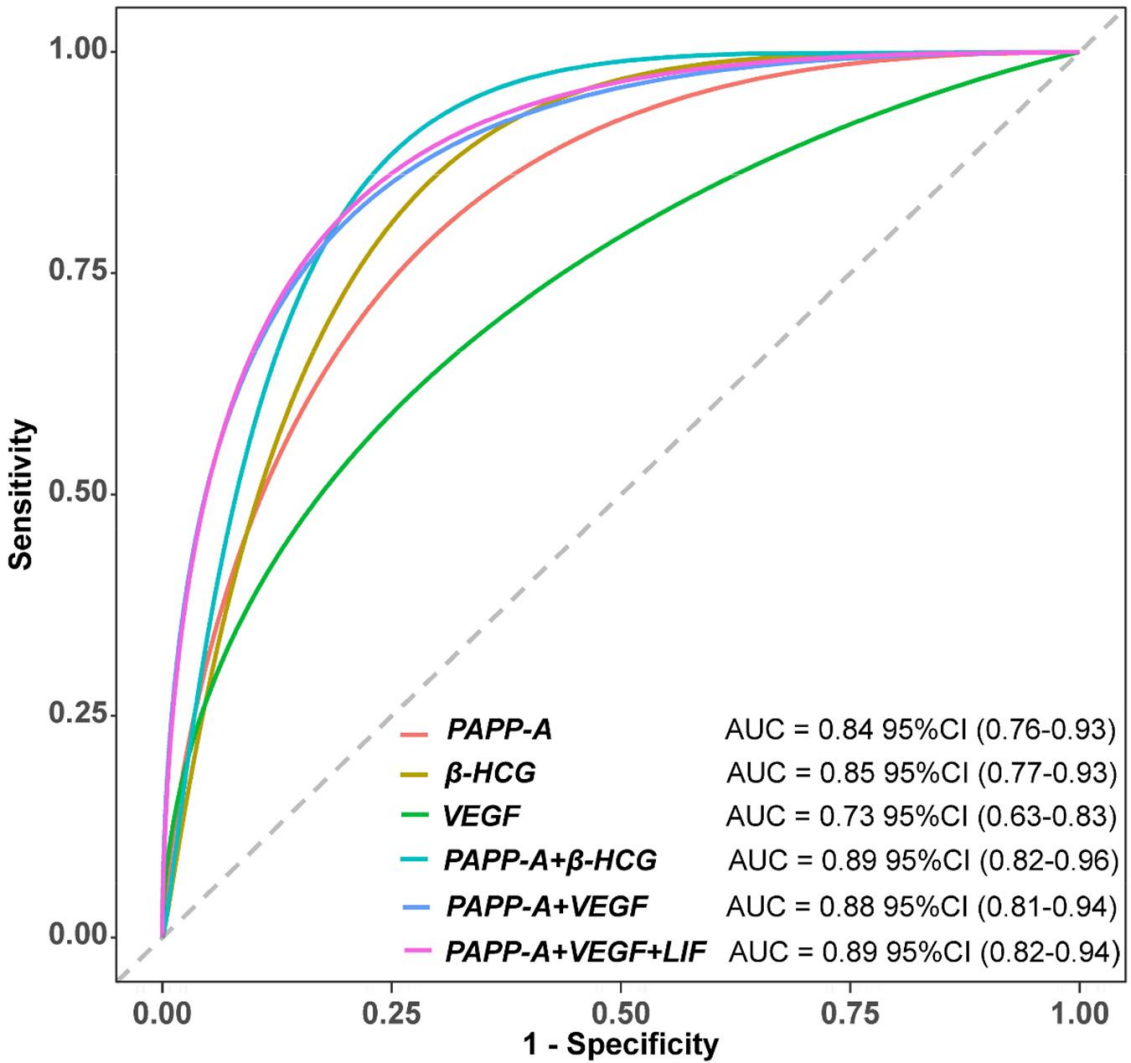
ROC curves for individual exploratory models of β-HCG, PAPP-A, VEGF, and LIF, and for combined exploratory models incorporating multiple biomarkers. AUC values and 95% confidence intervals are shown in the legend. All values are exploratory and derived from the same dataset used to construct the models.

Logistic regression models were applied to each biomarker individually, and ROC curves were derived from the predicted probabilities. As shown in Figure 2, the exploratory AUC values were: β-HCG 0.85 (95% CI: 0.77–0.93), PAPP-A 0.84 (95% CI: 0.76–0.93), VEGF 0.73 (95% CI: 0.63–0.83), and LIF 0.54 (95% CI: 0.41–0.64). The AUC values for β-HCG and PAPP-A were not significantly different (DeLong test, *p* = 0.87). The AUC of the combined model (PAPP-A + VEGF + LIF, AUC = 0.89) was significantly higher than that of VEGF alone (*p* = 0.001) and LIF alone (*p* < 0.001).

Exploratory thresholds, AUC, sensitivity, specificity values for each biomarker and combined model are summarized in Table 3. PAPP-A (exploratory threshold 8.13 ng/mL) showed an AUC of 0.84, sensitivity of 0.77, and specificity of 0.85, compared with β-HCG (threshold 678.5 mIU/mL; AUC 0.85, sensitivity 0.69, specificity 0.89) and VEGF (threshold 217.5 pg/mL; AUC 0.73, sensitivity 0.39, specificity 0.96). LIF showed the lowest performance (AUC 0.54).

**Table 3:** Exploratory performance of β-HCG, PAPP-A, VEGF, LIF, and combined models for distinguishing EP from normal intrauterine pregnancy.

Models	Exploratory threshold	AUC (95% CI)	Sensitivity (95% CI)	Specificity (95% CI)
β-HCG	678.5(mIU/mL)	0.85 (0.77–0.93)	0.69 (0.56–0.82)	0.89 (0.80–0.98)
PAPP-A	8.13 (ng/mL)	0.84 (0.76–0.93)	0.77 (0.65–0.89)	0.85 (0.74–0.95)
VEGF	217.5(pg/mL)	0.73 (0.63–0.83)	0.39 (0.26–0.53)	0.96 (0.90–1.00)
LIF	1,275 (pg/mL)	0.54 (0.41–0.64)	0.44 (0.30–0.58)	0.63 (0.49–0.77)
P + V		0.88 (0.81–0.94)	0.94 (0.87–0.99)	0.70 (0.56–0.83)
P + β‒HCG		0.89 (0.82–0.96)	0.79 (0.68–0.91)	0.91 (0.83–0.99)
P + V + L		0.89 (0.82–0.94)	0.77 (0.65–0.89)	0.87 (0.77–0.97)

All values are exploratory, derived from the same dataset used to build the models. Exploratory thresholds were identified using the Youden index. Combined models were constructed using multivariable logistic regression. All performance metrics should be regarded as hypothesis-generating only.

P represents PAPP-A, V represents VEGF, L represents LIF. CI, confidence interval.

### Associations between individual biomarkers and EP

As shown in Table 4, univariate logistic regression analysis revealed significant associations of PAPP-A (OR = 0.774, 95% CI: 0.692–0.849, *p* < 0.01), VEGF (OR = 1.014, 95% CI: 1.007–1.021, *p* < 0.01), and β-HCG (OR per 1,000 units = 0.855, 95% CI: 0.786–0.919, *p* < 0.01) with EP, while LIF (*p* = 0.414) showed no significant association. These models describe unadjusted associations and are not adjusted for potential confounders. Each 1-unit increase in VEGF was associated with a 1.4% higher odds of EP (OR = 1.014); each 1-unit increase in PAPP-A was associated with a 22.6% lower odds of EP (OR = 0.774); and each 1,000-unit increase in β-HCG was associated with a 14.5% lower odds of EP (OR = 0.855).

**Table 4:** Univariate logistic regression analysis of biomarkers for ectopic pregnancy.

Biomarkers	B	SE	OR	CI	Z	*p*
LIF	−0.002	0.001	0.998	(0.995–1.002)	−0.817	0.414
VEGF	0.014	0.003	1.014	(1.007–1.021)	3.746	<0.01*
PAPP-A	−0.256	0.051	0.774	(0.692–0.849)	−4.932	<0.01*
β-HCG/1,000	−0.157	0.039	0.855	(0.786–0.919)	−3.922	<0.01*

All models are unadjusted univariate analyses. These ORs describe exploratory unadjusted associations and relative changes in odds, and should not be interpreted as independent predictors or used for clinical decision-making.

* Statistically significant (*p* < 0.05).

CI, confidence interval; SE, standard error.

### Exploratory combined models

Combined multivariable logistic regression models incorporating multiple biomarkers were constructed to explore whether combinations might improve exploratory discrimination. As shown in Table 3, the combined AUC of PAPP-A and β-HCG (0.89; 95% CI: 0.82–0.96) and PAPP-A and VEGF (0.88; 95% CI: 0.81–0.94) were similar. The PAPP-A+ VEGF combination showed higher exploratory sensitivity (0.94) but lower specificity (0.70), while the PAPP-A+ β-HCG combination showed higher specificity (0.91) but lower sensitivity (0.79).

## Discussion

This exploratory case-control study characterized the associations between serum PAPP-A, VEGF, LIF, and β-HCG and EP in a single-center Chinese cohort, using a group of normal intrauterine pregnancies as controls. The main findings were: (1) median concentrations of PAPP-A and β-HCG were significantly lower in the EP group, while VEGF was significantly higher; (2) LIF did not differ significantly between groups; and (3) exploratory ROC analyses suggested that PAPP-A and β-HCG had comparable discriminatory performance (AUC 0.84 vs. 0.85), both outperforming VEGF and LIF in this comparison.

Prior studies have highlighted that reliance on β-HCG may lead to misclassification in approximately 17% of EP cases (10, 11). In the present study, β-HCG and PAPP-A demonstrated comparable exploratory performance, with AUC values of 0.85 and 0.84, respectively. These findings are broadly consistent with the prior work of Zhang et al. in a Chinese population, who reported an AUC of 0.81 for PAPP-A in distinguishing EP from normal intrauterine pregnancy (14). However, our results are inconsistent with those of Daponte et al., who found no significant difference in PAPP-A levels between EP and abnormal intrauterine pregnancy groups (20). This discrepancy is important to acknowledge and is likely attributable to the different control group: in contrast to our study, Daponte et al. used abnormal intrauterine pregnancies as controls. The inconsistent findings underscore that PAPP-A's ability to distinguish EP from normal intrauterine pregnancy may not reflect its ability to distinguish EP from the clinically more challenging differential diagnoses. More recently, Barnhart et al. showed that PAPP-A alone has only modest discriminatory performance in a multiplexed biomarker study, and high accuracy was achieved only when PAPP-A was combined with other biomarkers (11). This historical context reinforces the exploratory and hypothesis-generating nature of the present findings.

Recent investigations have further established associations between low PAPP-A levels and adverse pregnancy outcomes, including miscarriage, premature birth, and pre-eclampsia (21). The integration of PAPP-A with other biomarkers, such as AFP and uE3, has been shown to offer potential for early clinical assessment (22). The reduced PAPP-A levels observed in EP in this study may reflect insufficient trophoblastic development, an abnormal implantation environment, or restricted formation of early placental-like tissue. However, the exploratory threshold of 8.13 ng/mL identified requires validation in independent prospective cohorts.

Regarding VEGF and LIF: our findings demonstrated that VEGF levels (187.5 vs. 126.5 pg/mL) were significantly elevated in the EP group compared with the control group, whereas LIF (984 vs. 987.1 pg/mL) did not differ significantly. The exploratory AUC of VEGF was 0.73, with low sensitivity (0.39) but high specificity (0.96) at the threshold. Prior studies have reported higher VEGF concentrations in patients with EP vs. normal or submolar intrauterine pregnancies, with a threshold of 200 pg/mL achieving 88% sensitivity and 100% specificity (23). Other studies have reported sensitivity and specificity of 96.7% and 95.0%, respectively (15). The lower sensitivity observed in our study may reflect differences in control group selection and assay methodology. These findings suggest that the value of VEGF alone in differentiating EP from normal intrauterine pregnancy is limited in this setting, and that combined approaches may provide better exploratory discrimination.

Univariate logistic regression analysis demonstrated significant unadjusted associations of PAPP-A, VEGF, and β-HCG with EP. It is important to note that in this exploratory case-control study, these logistic regression models describe the unadjusted associations and changes in relative risk ratios, rather than independent effects or validated clinical predictions. The potential confounding factors, such as previous endometriosis, pelvic inflammatory disease, history of infertility, history of using assisted reproductive technology, and history of previous fallopian tube surgery, were not included in the multivariate model. The observed associations may therefore reflect underlying risk factors rather than direct biomarker relationships with EP.

Several limitations of this study warrant careful consideration. First, the case-control design introduces inherent limitations. The selection of clearly defined EP cases and healthy intrauterine pregnancy controls creates an “extreme phenotype” comparison that is likely to overestimate biomarker performance compared with the real clinical challenge, which arises in the PUL population. The retrospective nature limits our ability to establish temporal relationships and to control for all relevant confounders. Second, the control group was limited to patients with normal intrauterine pregnancies, which does not reflect the most clinically challenging differential diagnosis. Performance in this more relevant clinical context is unknown. Third, the relatively small sample size (48 EP cases, 46 controls) increases the probability of imprecise estimates and overestimated performance due to overfitting; results should be interpreted with caution. Fourth, residual within-group variation in gestational age (6–8 weeks) could not be fully controlled, and the potential confounding effect of gestational age on PAPP-A levels could not be reliably assessed due to sample size constraints. Fifth, several established EP risk factors (previous EP, pelvic inflammatory disease, assisted reproductive technology, prior tubal surgery, infertility) were not incorporated into multivariate analyses, precluding adjustment for confounding and meaningful subgroup analyses.

Future research should prioritize prospective cohort designs enrolling patients presenting with early pregnancy symptoms or PUL, which would enable a more rigorous and clinically relevant assessment of whether PAPP-A and other biomarkers can provide additional value for clinical decision-making in the most needed “grey zone” of diagnosis. Large-scale, multicenter prospective studies incorporating multivariate logistic regression for confounding adjustment, gestational age as a key covariate, and formal sample size calculations based on the effect estimates from this exploratory work are essential to confirm and extend these findings.

Taken together, the present data provide preliminary, hypothesis-generating evidence that PAPP-A may serve as a candidate biomarker for the early detection of EP, with exploratory performance comparable to β-HCG in an extreme-phenotype comparison with normal intrauterine pregnancy. This work follows the logic of an exploratory case-control study and is aligned with STROBE recommendations. A future, dedicated diagnostic accuracy study following the STARD (Standards for Reporting Diagnostic Accuracy Studies) guidelines, conducted prospectively in clinically relevant populations, is required to formally evaluate the clinical utility of PAPP-A.

## Data Availability

The raw data supporting the conclusions of this article will be made available by the authors, without undue reservation.
